# The Use of Laminates of Commercially Available Fabrics for Anti-Stab Body-Armor

**DOI:** 10.3390/polym13071077

**Published:** 2021-03-29

**Authors:** Nguyen Quang Khuyen, Phan Vu Duc Han, Ngoc Tuan Nguyen, Quoc Bao Le, Madis Harjo, Gholamreza Anbarjafari, Rudolf Kiefer, Tarmo Tamm

**Affiliations:** 1 Conducting Polymers in Composites and Applications Research Group, Faculty of Applied Sciences, Ton Duc Thang University, Ho Chi Minh City 700000, Vietnam; nguyenquangkhuyen@tdtu.edu.vn (N.Q.K.); lequocbao@tdtu.edu.vn (Q.B.L.); 2 Faculty of Applied Sciences, Ton Duc Thang University, Ho Chi Minh City 700000, Vietnam; phamvuduchan1@gmail.com (P.V.D.H.); nguyenngoctuan@tdtu.edu.vn (N.T.N.); 3 Intelligent Materials and Systems Lab, Institute of Technology, University of Tartu, Nooruse 1, 50411 Tartu, Estonia; madis.harjo@gmail.com (M.H.); tarmo.tamm@ut.ee (T.T.); 4 iCV Research Lab, Institute of Technology, University of Tartu, 51009 Tartu, Estonia; shb@ut.ee

**Keywords:** linen, soft and hard silk, laminates, adhesives, shore hardness

## Abstract

Modern personal protective armor has been generally based on the Kevlar fabrics, with the main goal to offer defense against bullets. In addition to the high cost and poor processability, Kevlar has the disadvantage of limited stab-proofing capability. On the other hand, a large number of crimes involving deadly injures represent knife attacks. Our goal in this work was to investigate composites based on traditional commercially available fabrics of linen and silk, using different adhesives-polymers for forming laminates. The silk composites also contained different amounts of in-woven polyester. Three different water-based adhesives of polyurethane, urea formaldehyde and polyvinyl alcohol were considered. It was found, that besides the strength of the fabrics themselves, the adhesives polymers played a crucial role in the obtained performance of the laminates. The laminates were characterized in their mechanical properties, as well as with scanning electron microscopy and FTIR spectroscopy.

## 1. Introduction

Protective body-armor has a long history, as protection against arrows, spears and knives in battles was used in ancient times [1]. The application of linen laminates dates back 3000 years ago to the Egyptian empire, where lightweight, wearable and easy formable body-armor was used [1]. Contemporary studies [2] of linen laminates fabricated using ancient techniques and materials like linen seed adhesives have found that a certain protection against knives and arrows could indeed be obtained through their use. In ancient Asian cultures, body-armor made from silk was used and further laminated with silver plates [1], making the armor easily wearable and lightweight. Today, protective body-armor for police and military forces is largely based on Kevlar fabrics [3], with the main goal to protect the wearer from bullets. In most cases, pure Kevlar fabrics do not possess stab-proof properties, but composites with linen have shown reasonable stab-proofing [4], with silica-coated Kevlar wool being another option showing good results [5]. Perhaps more so than bullet penetration, stab-penetration is a function of many parameters, including blade sharpness and geometry, cutting angle and velocity, etc. [6]. While knives of different characteristics and quality can lead to different performance, the general process from indentation/puncture to penetration is the same. Stab and punctuation resistance depends on the relative interaction strength of friction of the yarns (fabrics) and the knife, as well as inter-yarn frictions [7]. Addressing these aspects, attempts have been made to influence the friction by adding shear thickening non-Newtonian fluids to composites [8]. Various different fabrics have been introduced in the literature [9] to obtain stab proof properties [10] with different designs. 

Unlike in the USA, where firearms can be purchased relatively freely, in most countries the use of weapons is strictly controlled. Most criminal attacks in those countries are driven by stabbing while employing sharp objects such as knives, leaving victims heavily injured if not dead [11,12]. Body-armors designed and produced for the military or law-enforcement are expensive and not readily available for the general public. Our motivation for this work was to analyze whether much cheaper alternatives could be produced from commercially available fabrics and adhesives. There have been very few attempts using commercially available fabrics, including a study on linen leaning on history [2] or triaxially woven fabrics [13]. 

Therefore, our goal in this research was to bring the concept of textile-based body-armor from history to the present era, while applying commercially available fabrics of linen and silk blends (hard silk with 50% in-woven polyester, soft silk with 20% in-woven polyester) forming novel laminates with different adhesives and in number of layers. The shore hardness and tensile strength on the laminates was studied to find the best designs. 

SEM images of linen, soft silk and hard silk with and without the adhesives were recorded, and FTIR spectroscopy of the composites was carried out, to evaluate the adhesion properties of different adhesives on the fabrics.

## 2. Material and Methods

### 2.1. Materials

Linen (flax fiber, plain weave, woven, 130 GSM, 0.15 mm thickness) and silk blends of soft silk with 20% polyester (plain weave, woven, 107 GSM, 0.21 mm thickness) and hard silk with 50% polyester (plain weave, woven, 129 GSM, 0.26 mm thickness) were purchased from Thanh Cong Textile Co. Ltd., Ho Chi Min City, Vietnam, and used as obtained. Polyurethane (Best-PU-1504, two component base A to hardener B 1:2 weight %, (Best Klebstoffe GmbH & Co. KG, Kinsau, Germany) component A (base) density 1.0 g cm^−3^, viscosity 1300–3500 CPS, component B (hardener) density 1.17 g cm^−3^, 3300 CPS, after mixing of A and B viscosity 50,000 CPS, working time 1–5 min) urea formaldehyde (UF, 25 wt.%, An Thai Khang JSC, HCMC, Vietnam, dog glue X-66, viscosity 6500 CPS, density 1.3 g cm^−1^, working time 5 min) and polyvinyl alcohol (PVA, 11–12%, U.R. URAI Paints Co. Ltd., HCMC, Vietnam, white glue or ATM, density 1.27 g cm^−3^, viscosity 13,000 CPC, working time 5 min) were used as supplied.

### 2.2. Methods 

The lamination process was performed in in Kim Cuong Production Trading Service Joint Stock Company (Ho Chi Minh City, Vietnam). The different fabrics were impregnated with adhesives (at room temperature) using templates to cover the required areas for the various samples on the 25 cm × 25 cm fabric pieces. The adhesive-covered fabrics were faced together. The process was repeated until the desired number of layers (2–20) was achieved. The laminate stacks were cured at 60 °C for 24 h at a pressure of 5 MPa under a custom hydraulic press with a controlled temperature. The residual fabric from around the samples was cut away, and three samples for each size-material were fabricated. The weights and fabric-adhesive weight ratios of the fabricated 20-layer laminates are listed in Table 1. The variations in the weight ratios were due to the differences of adhesive densities, viscosity and also wetting of the fabrics.

Bonding and tensile strength of the composites in dimension of 2.54 cm width and 10 cm length were carried out following the ASTM F904 (XLW (PC)) and ASTM D638 standards (ASTM International, West Conshohocken, PA, USA), respectively. The Grips method—a gripping system that consists of a fixed bottom grip and an upper grip assembled with a pulling system—was applied. This system would pull up until adhesive failure is caused in the material—failure occurs at the interface of two adjacent layers [12]. An Auto Tensile Tester device (Labthink Instrument Co., Ltd., Medford, MA, USA) was used for the experiments [14]. The young′s modulus was determined from the strain-stress measurements. The strain ε was calculated over the length of the material after stretching L in relation to the original length L_0_ of the fabrics (ΔL = L − L_0_).

The force F was given in Newtons (1N = 1 kg m s^−2^) divided by the surface area A (width * thickness) of the samples.

The shore D hardness (HA, hardness) of the laminates was determined on the surface using a shore D durometer (Digital Shore D Durometer High Hardness Rubber Tester 100HD, Zhengzhou Nanbei Instrument Equipment Co., Ltd., Zhengzhou, China). A Manuel test of knife penetration performed by an adult male was applied using the German standard test NJ 0115.0 [15]. Bullet proof Manuel testing was performed using a 5 mm Remington Rimfire Magnum pistol (Remington Arms Company, LLC, Madison, NC, USA). 

The fabrics of linen, soft and hard silk with and without adhesives were investigated by SEM (VEGA TESCAN, TESCAN ORSAY HOLDING, Brno-Kohoutovice, Czech Republic) and FTIR spectroscopy (Bruker Alpha, with Platinum ATR, Billerica, MA, USA).

## 3. Results and Discussion

### 3.1. Influence of Adhesives on Fabrics

**Scheme 1:**
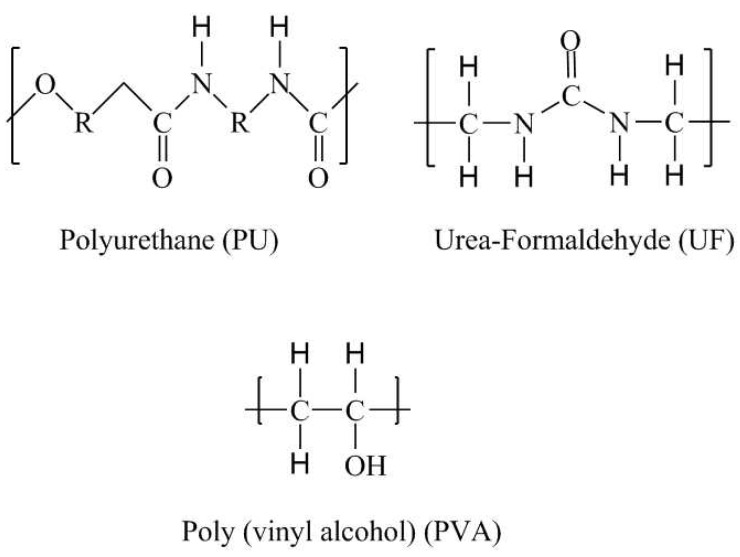
The chemical structure of the adhesives of polyurethane (PU), Urea-Formaldehyde (UF) and poly (vinyl alcohol) (PVA).

The chemical structures of the applied adhesives are shown in Scheme 1, whereas the reaction of adhesives on different fabrics are discussed in their mechanism, as well their advantages and disadvantages.

In the structure of PU (Scheme 1), the group “R” can be ethylene units or other olefins which determine the flexibility of the polymer [16]. PU adhesives can be fairly elastic, leading to relatively flexible laminates of linen and the silk blends. Depending on the ratio of the base polymer and the curing agent, the elasticity-rigidness can be tuned, with higher curing agent concentration resulting in stronger, but also stiffer, more rigid laminates. The curing was performed at 60 °C for 1 day. 

**Figure 1:**
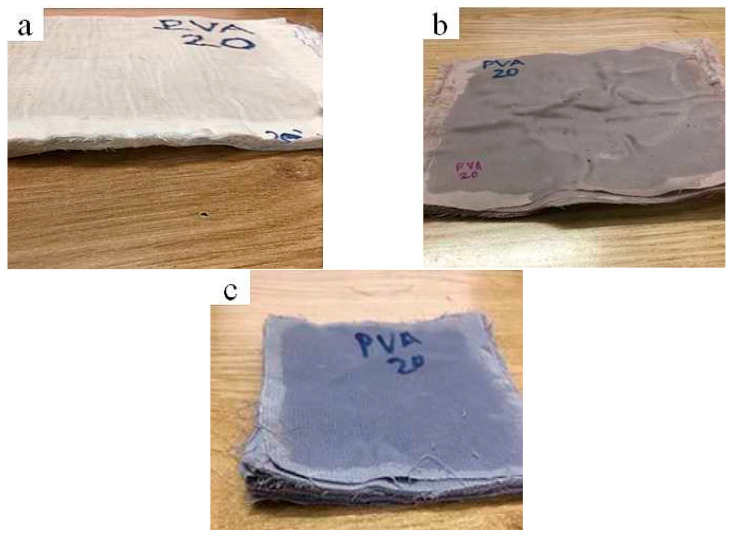
Laminates (20 layers) using PVA adhesives on (**a**) linen, (**b**) soft silk and (**c**) hard silk.

The main application for UF adhesives is cellulose materials such as wood. PVA adhesive, also called “white glue” or milk glue” due to its color being applied largely in the textile industry [17]. It has an unpleasant smell but has shown no acute toxicity [18]. The disadvantages of PVA applied on linen or silk laminates are the long curing times at room temperature or even at 60 °C for two days. The main issue of PVA is the high amount of water released during curing, which affects the laminates forming wrinkled surfaces, as shown in Figure 1a–c.

Laminates of linen and soft silk with PVA adhesives were most affected by wrinkling (Figure 1a,b). Hard silk did not show any wrinkles on the surfaces, which is likely the effect of 50% in-woven polyester making hard silk more hydrophobic than the other materials, and thus, reducing the humidity effects during curing.

#### SEM Images

**Figure 2:**
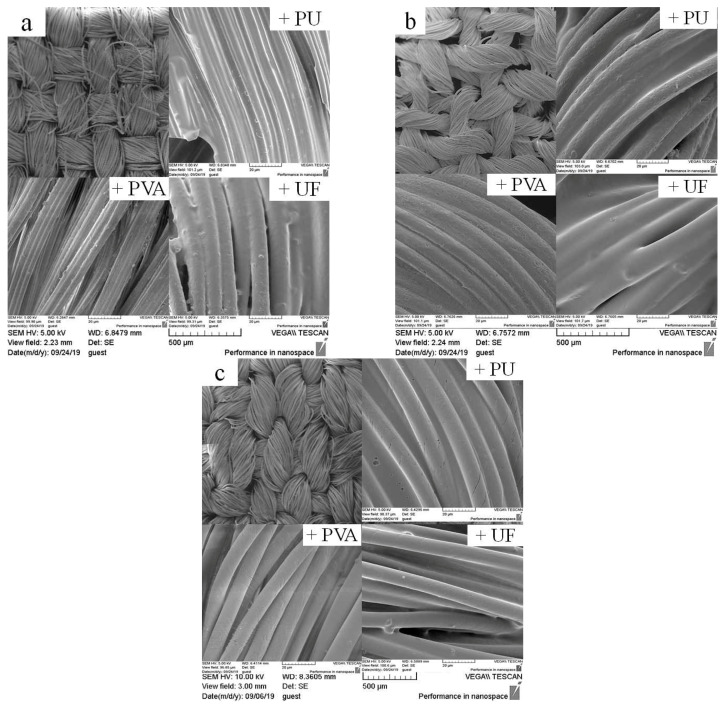
SEM images (scale bar 500 μm) with insets of adhesive impregnations with PU, PVA and UF (scale bar 20 μm) (**a**) linen, (**b**) soft silk and (**c**) hard silk.

The structure of the fabrics of linen, soft silk and hard silk with and without impregnation are shown as SEM images in Figure 2.

In order to avoid differences from the weave, all fabrics were chosen with pain weave, showing the most common industrially applied regular structure, as expected [19]. When comparing the plain weave structures of linen (Figure 2a), soft silk (Figure 2b) and hard silk (Figure 2c), hard silk had the densest and most compact weave, followed by linen with some gaps and more space in the wave of soft silk. The intransitive effects can be observed in the insets in Figure 2a–c, indicating that apart from the UF-impregnated hard silk, other adhesive-fabric combinations resulted in fairly uniform coatings with individual filaments found to be intransitive together. In case of linen and PVA, separation of tightly intransitive-together bundles could be observed, whereas hard silk was clearly less coated with PVA. The main reasons for more film like adhesives in soft silk and linen were attributed to a looser weave, leaving more space between individual filaments, which could be filled with adhesive by capillary forces. However, tighter-denser weaves in general make for stronger fabrics and also stronger laminates. 

**Figure 3:**
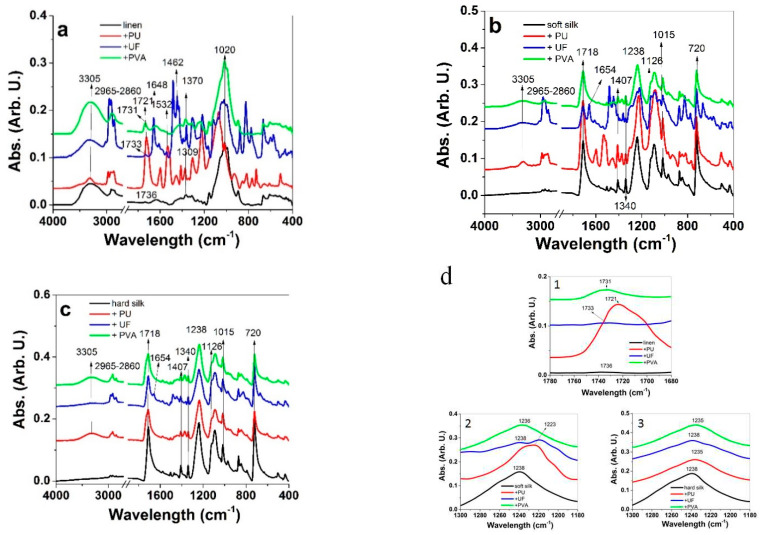
FTIR spectra (4000–400 cm^−1^) of the fabrics (**a**) linen, (**b**) soft silk and (**c**) hard silk without (black) and with the addition of the adhesives: PU (red), UF (blue) and PVA (green). The magnification of peaks in (**d**) are shown in for linen (1) at the 1736–1721 range and for the soft silk (2) and hard silk (3) with adhesives PU, UF and PVA at 1238 cm^−1^.

To investigate the interactions between the adhesives with the fabrics, FTIR measurements were performed, with the results presented in Figure 3a–c.

Linen consists of flax fibers, which are mainly made up of cellulose with 16.7% hemicellulose 1.8% pectins, 2% lignin and 1.5% waxes and fats. Typical peaks of the fabrics and the adhesives (Figure 3a–c) are listed below, starting from 3305 cm^−1^ of polar hydrogens (O-H and N-H) stretching vibrations from the fabrics, the adhesives and the water molecules. The peaks between 2965 cm^−1^ to 2860 cm^−1^ represent C-H and CH_2_ stretching vibrations of linen [20] and UF adhesive [21], as well as CH_3_ groups from PU [22] and PVA (Figure 3a–c). In Figure 3a, the small 1736 cm^−1^ peak for linen [20] and with PU, UF and PVA represented C=O groups stretching vibrations found in range of 1721–1736 cm^−1^. The 1648 cm^−1^ peak from UF adhesives represents the amide I group, the amide II is located at 1522 cm^−1^ and the amide III at 1462 cm^−1^ [21]. The linen specific symmetrical deformation of CH_3_ groups from lignin [20] are shown at 1370 cm^−1^ with shifts for UF adhesive to 1364 cm^−1^. The strong peak at 1020 cm^−1^ can be identified as C-O-C bending vibration of cellulose units in linen. 

The spectra of silk fibroin blended with 20% in-woven polyester are shown in Figure 3b. The 1718 cm^−1^ sharp peak represents C=O stretching vibration, 1407 cm^−1^ the aromatic ring vibration [23], 1340 cm^−1^ the CH_2_ wagging, a shoulder at 1126 cm^−1^ of the asymmetric stretching vibration of C-O-C groups [24] and the 720 cm^−1^ peak shows the C-H vibration of the aromatic ring [23] in polyester. The silk fibroin typical signals can be found at 1238 cm^−1^ identified as amide III peak [25] stretching vibration while the 1015 cm^−1^ peak refers to the C-C stretching vibration [26]. The C=O groups of the amide I and II can be identified between 1654–1540 cm^−1^ [27]. In case of PU adhesive, a sharp peak at 1532 cm^−1^ can be identified as amide II vibration of N-H and C=N and a shoulder at 1310 cm^−1^ presents the amide III peak [28]. Figure 3c shows the spectra of hard silk where polyester was 50% in-woven, which can be well observed in the more dominant polyester peaks at 1718 cm^−1^, 1407 cm^−1^, 1340 cm^−1^ and 1126 cm^−1^ and 720 cm^−1^ in comparison to soft silk (20% polyester) in Figure 3b. The peaks for silk fibroins of 1238 cm^−1^ and 1015 cm^−1^ seen in Figure 3b are shown in reduced intensity in Figure 3c. 

The interactions between the fabric materials with those of the adhesives could be observed from the shifts of the relevant FTIR signals shown in Figure 3d by exploring the 1736–1721 cm^−1^ (C=O stretching) of linen with different adhesives as well as the 1238 cm^−1^ (amide III) of silk fibroins [29]. It is well known that shifts to lower wavelength identify stronger hydrogen bonds, while shifts to higher wave length hint towards weaker hydrogen bonds [30]. In the case of linen (Figure 3d,1) the shift of the carbonyl group from 1736 cm^−1^ for linen to 1721 cm^−1^ with PU, to 1733 cm^−1^ with UF and to 1731 cm^−1^ with PVA was seen. While all of the adhesives are capable of hydrogen bonding, the strongest shift was observed for PU, whereas it is well-known that PU forms strong hydrogen bonds with flax fibers [30]. In case of silk fibroins, the typical amide III signal at 1238 cm^−1^ for soft silk (Figure 3d,2) also showed the biggest shift for PU (to 1223 cm^−1^) followed by PVA (1236 cm^−1^) while UF showed no shift. In case of hard silk, with higher polyester content, the hydrophobicity of the composite increased and there were only very minor shifts of the 1238 cm^−1^ amide III peak observed for PU and PVA at 1235 cm^−1^. Besides hydrogen bonds, additional hydrophobic interaction in fibroin silk can lead to inter- and intra-molecular interactions [31], whereas the higher amount of in-woven polyester in hard silk will enhance the hydrophobic interactions. Therefore, in contrast to the other fabrics, the interactions of hard silk with the adhesives were dominated by non-specific van der Waals bonds [32].

Another effect to consider is related to the polar hydrogen atom related peaks in the 3300–3500 cm^−1^, region. For pure fabrics, there was a strong absorption for the hydrophilic (and O-H possessing) linen, while no peak is observed for the silks. Among the adhesives, the spectra of PU can be distinguished, where the narrower peaks are likely related to the N-H groups without much contribution from water or other hydrogen-bonding interactions. As expected, the strongest—broad and tall—peaks stem from the interactions of the most hydrophilic materials: linen and PVA. The increasing impact of the less hydrophilic polyester in the silk blends reduces the intensities of these peaks considerably (Figure 1c). 

### 3.2. Material Properties

**Figure 4:**
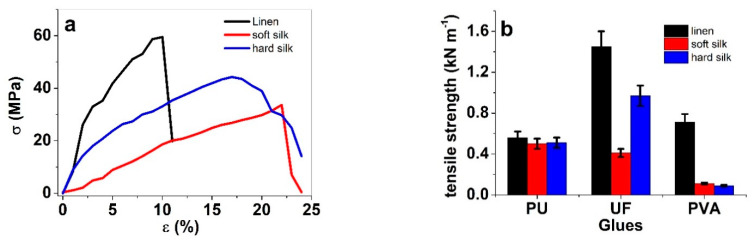
Sheets of linen (black), soft silk (red) and hard silk (blue) showing in (**a**) strain-stress curve, and in (**b**) tensile bond strength applied with different adhesives (PU, UF and PVA).

The strength of the laminate is a combination of the properties of the fabrics, the adhesives and the interactions. The properties of the fabrics as single sheets (repeated with three different samples, results shown as mean values in Figure 4b) were studied and compared. The strain stress curves are shown in Figure 4a. The interaction strength was characterized by tensile bonding strength measurements (Figure 4b).

The young′s modulus of the fabric determined from the slope of the first linear section of the stress-strain curves (Figure 4a). The stiffest fabric with the highest modulus of 18 ± 1.2 MPa was the linen, followed by hard silk with 7.2 ± 0.5 MPa and soft silk with just 1.4 ± 0.1 MPa. The latter also showed the largest elongation of 23 ± 2%. Therefore, while our linen was clearly the stiffest and strongest, the relatively large strains up to 10 ± 1% were somewhat unexpected, as linen yarn has been reported to be just 2% stretchable [33], while the reported young′s modulus of 1.6 Gpa was also not quite comparable. This is likely because combining the yarns into fabrics lowers the stiffness and increases strain. Moreover, fabrics from natural fiber yarns are prone to aging effects, accompanied by decreasing modulus [33], complicating comparisons. 

The strongest bonding of any fabrics with the different adhesives (Figure 4b) was achieved by intransitive linen with UF (1.45 ± 1.6 kN m^−1^). The second-best tensile strength was for hard silk fabric with the same UF adhesive (0.97 ± 0.1 kN m^−1^). Linen was also strongly bonded with PVA (0.71 ± 0.08 kN m^−1^). Interestingly, PU appeared to be indifferent to the fabric type, as all three fabrics had nearly identical results. 

#### 3.2.1. Shore Hardness of Laminates

**Figure 5:**
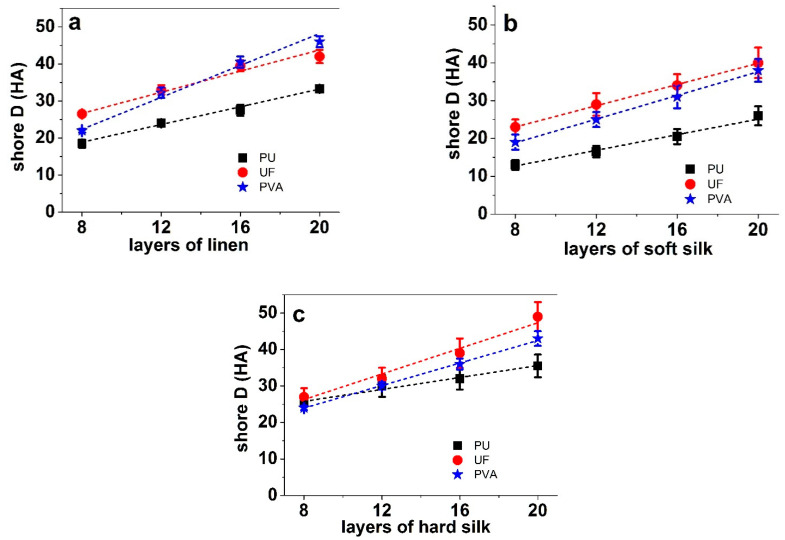
Shore D hardness (HA) of textile laminates with different adhesives: PU (■, black), UF (●, red) and PVA (★, blue) and fabrics 8–20 layers; (**a**) linen, (**b**) soft silk and (**c**) hard silk. The dashed lines represent the linear fits and are shown here for orientation only.

The primary characteristic of a material to be applicable as anti-stabbing body-armor must be resistance against local plastic deformation—indentation. There is a variety of different hardness scales to assess this property, the shore D hardness scale was chosen here based on the materials. The hardness value is determined by the indentation depth of the durometer probe into the sample powered by a specified force of a loaded spring. The hardness of the material is then given as a dimensionless HA (S) value with larger values, indicating higher hardness. As a reference, in a recent study the hardness of a composite combining Kevlar and glass fiber with cotton fabrics was determined to reach shore D 79 [34], which should be enough to offer protection against bullets and stabs. However, the high price and difficult processability of Kevlar sets limits to the potential of wide-spread affordable protection. The shore D hardness values determined for laminates of 8–20 layers (below 8 layers, the thickness was not enough for durometer measurements) of linen, soft silk and hard silk and the different adhesives are shown in Figure 5.

The hardness increased almost linearly with the increasing number of layers, however, the slopes of the dependencies were not equal. In case of linen fabrics, the hardness of PVA composites had a steeper slope and reached the highest value of shore D 46 ± 2.5 by 20 layers, followed by 42 ± 2.8 for UF adhesive. UF was also the best adhesive for both types of silks, with hardness values reaching 40 ± 3.5 and 49 ± 4.1 by 20 layers for soft and hard silk, respectively. PVA was a close second for both silks. The PU adhesive had the lowest hardness values for any fabric, which agrees well with the above discussed interaction and bonding results. The hardness values for UF and PVA on hard silk and linen, respectively, compare favorably with a recent study [4], where Kevlar and linen composites with PVA reached a shore D value of 40. The properties of the laminates of 20 layers of linen, soft silk and hard silk with PVA, UF and PU adhesives are compared in Table 2. 

The data in the table show that while overall the hard silk composites with UF present the highest values, if the thickness is also considered, the linen-PVA composites stand out as well, with just slightly lower hardness and Young’s modulus values, but also at lower overall thickness. There is virtually no correlation between hardness and bonding strength.

#### 3.2.2. Stab-Proof and Bullet-Proof Tests

**Figure 6:**
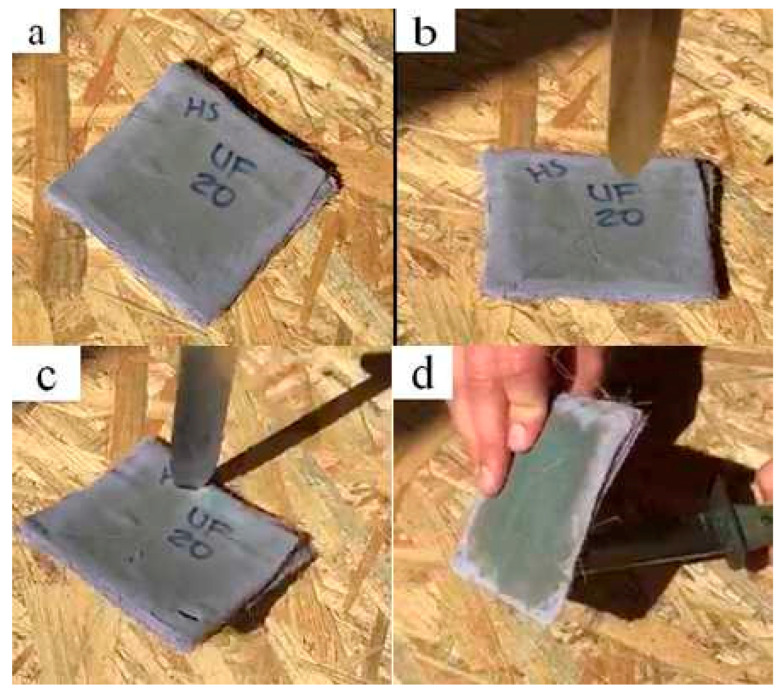
Manual stabbing test. (**a**) the laminate HS (20 layers, UF adhesive), (**b**) the knife position before stab, (**c**) the stab into the fabric and (**d**) the backside showing no penetration of the blade.

While the various material characteristics are important for stab-resistance, the true performance of a material has to be tested also in a relevant scenario. As there is no “standardized stabbing” in real world, a manual test of knife penetration according to the Germany standard test NJJ 0115.0 was performed, where an average male adult is stabbing the studied material, with a typical maximum energy of 33 J in level 2 [15]. As the laminates with the highest hardness and stiffness were those with the UF adhesive, the tests were carried out on 20-layer UF laminates (repeated on three different samples) shown as images of one sample in Figure 6.

While the soft silk and linen laminates failed the test as they were penetrated by the blade, the laminate of hard silk had a penetration of just 3 ± 0.2 mm (Figure 6), which meets the requirements for stab-protective vests. To find out how far the linen and soft silk laminates were from passing, a round of tests was carried out with double-thickness laminates (40 layers). Both soft silk and linen with UF adhesive showed a penetration depth of 8 ± 0.7 mm, which explains the failure of the 20-layer laminates. As the typical protective vest on the market are in thickness range of 7 mm–10 mm, the hard silk—UF laminate with a thickness of 7.9 mm showed clear promise in this study. Surprisingly, there was little difference in the performance of linen and soft silk, while all the measured characteristics had predicted poorer performance for soft silk.

While it was not the primary goal of the study, additional tests of bullet proofness were carried out on the laminates using 5 mm Remington Rimfire Magnum pistol shooting from a 15 m distance. As expected, none of the laminates were able to stop any bullets, even in double or triple arrangements. Therefore, while the common fabric-based laminates cannot offer protection against bullets, stab-resistance is achievable. Further experiments with different types of knives, other sharp objects and slashing impacts need to be carried out to verify the performance and optimize the composites [35].

For potential applications in stab-proof vests, in case of PVA the water-resistance should be considered and potentially improved with an additional coating layer. PU and UF, as thermoset polymers, should not have significant issues; however, the hardness and stab-proofing under different humidity/wetness conditions deserves further studies. The breathability of the laminates is another question that needs attention when actual wearable items are being designed in the future.

## 4. Conclusions

Even in ancient times, body-armor based on laminated fabrics offered protection against stabbing or other weapons. In an attempt to bring this traditional approach into modern age, commercially available fabrics were laminated into composites using three different types of adhesives. As expected, both the fabric type and the nature of the adhesives had an effect on the properties of the laminates, like tensile strength, hardness and bonding strength. While for linen fabrics, the PVA adhesive gave the best result, it also showed a drawback of wrinkling and deformation during fabrication, likely due to high hydrophilicity. The laminates with the UF adhesive showed higher flexibility while reaching the highest hardness (shore D up to 49). The manual stab tests showed that a 20-layer hard silk laminate with UF adhesive with a 7.9 ± 0.3 mm thickness level can prevent level 2 stabs, resulting a knife penetration of 3 ± 0.2 mm. Further research and optimization are needed to study other aspects of the stab-resistance, the final envisaged material could provide a low cost form of protection against stabbing, which could be of interest especially in countries where the fatalities due to knife attacks is a rising problem, such as Vietnam, Ireland or the UK.

## Figures and Tables

**Table 1:** Composition of 20 layers of linen, soft silk and hard silk laminates with different adhesives: weight (of 20 × 20 cm piece) and weight ratios of fabrics: adhesives.

20-Layer Laminates	Weight (g)	Weight Ratio (Fabric:Adhesive)
Linen		
PVA	209 ± 9	1:1
UF	187 ± 12	1.2:1
PU	160 ± 12	1.8:1
Soft-silk		
PVA	174 ± 12	1:1
UF	179 ± 13	1:1.1
PU	153 ± 7	1.3:1
Hard-silk		
PVA	164 ± 11	1.7:1
UF	237 ± 14	1:1.3
PU	230 ± 15	1:1.2

**Table 2:** Comparison of linen, soft silk and hard silk 20 layers laminates with different adhesives: thickness and hardness.

20 Layer Laminates	Thickness (mm)	Hardness Shore D (HA)
Linen		
PVA	5 ± 0.3	46 ± 2.5
UF	4.6 ± 0.2	42 ± 2.8
PU	4.2 ± 0.3	33 ± 2.0
	Soft silk	
PVA	5.9 ± 0.4	38 ± 2.8
UF	6 ± 0.4	40 ± 3.5
PU	5.6 ± 0.3	26 ± 1.5
	Hard silk	
PVA	6.4 ± 0.5	43 ± 3.9
UF	7.9 ± 0.5	49 ± 4.1
PU	7.7 ± 0.6	35 ± 3.1

## Data Availability

The data presented in this study are available on request from the corresponding author.
